# A Comparison of Masculinity Facial Preference Among Naturally Cycling, Pregnant, Lactating, and Post-Menopausal Women

**DOI:** 10.1007/s10508-017-1093-3

**Published:** 2017-10-25

**Authors:** Urszula M. Marcinkowska, Grazyna Jasienska, Pavol Prokop

**Affiliations:** 1 Department of Environmental Health, Faculty of Health Sciences, Jagiellonian University Medical College, 31-531 Cracow, Poland; 2 Faculty of Education, Trnava University, Trnava, Slovakia; 3 Institute of Zoology, Bratislava, Slovakia

**Keywords:** Facial preferences, Fertility, Post-menopausal, Pregnancy, Sexual dimorphism

## Abstract

Women show cyclical shifts in preferences for physical male traits. Here we investigated how fertility status influences women’s facial masculinity preference in men by analyzing a large sample of heterosexual women (*N* = 3720). Women were regularly either cycling (in both low- and high-conception probability groups), lactating or were currently in a non-fertile state (pregnant or post-menopausal). Analyses simultaneously controlled for women’s age and sexual openness. Participants via two alternative forced choice questions judged attractiveness of masculinized and feminized men’s faces. After controlling for the effect of age and sociosexuality, regularly cycling and pregnant women showed a stronger preference for masculinity than lactating and post-menopausal women. However, there was no significant difference in masculinity preference between women in the low- and high-conception probability groups. Women’s sociosexuality showed a positive, but very weak association with men’s facial masculinity preference. We suggest that women’s overall, long-term hormonal state (cycling, post-menopausal) is a stronger predictor of preference for sexual dimorphism than changes in hormonal levels through the cycle.

## Introduction

### Facial Masculinity

Men’s facial sexual dimorphism is related to their perceived attractiveness (Gangestad & Scheyd, 2005). Facial masculinity is positively associated with men’s health (Rhodes, 2006; Thornhill & Gangestad, 2006), immunity (Rantala et al., 2012), dominance and competitive ability (Archer, 2009), attractiveness (Dixson, Sulikowski, Gouda-Vossos, Rantala, & Brooks, 2016), and overall mating success (Rhodes, Chan, Zebrowitz, & Simmons, 2003). On the other hand, less masculine men can be judged attractive due to their perceived amenableness to women and look like providers who invest in their offspring (Dixson, Tam, & Awasthy, 2013). Until now, there is no agreement on whether women prefer more feminine or more masculine men, or show enhanced preference for either (Burriss, Marcinkowska, & Lyons, 2014; Perrett et al., 1998; Peters, Simmons, & Rhodes, 2008; Zietsch, Lee, Sherlock, & Jern, 2015). It is now clear, however, that there is no stable, common preference shared by all women throughout their lifetimes. High masculinity is suggested to correlate not only with good genes and health, but also with some undesired personality traits, less interest in long-term relationships, or lower paternal investment (Boothroyd, Jones, Burt, & Perrett, 2007; Kruger, 2006; Perrett et al., 1998). Thus, women’s overall preferences for highly sexually dimorphic males can be a result of a trade-off between positive and negative effects of high masculinity. For example, preferences for facial masculinity increase when rating men for short-term relationships rather than long-term (Little, Connely, Feinberg, Jones, & Roberts, 2011) or when women judge putative partners for extra-pair sexual relations (Penton-Voak et al., 1999).

On the other hand, Boothroyd et al. (2017) showed that intermediate, rather than high, levels of men’s masculinity were associated with offspring survival, which does not support the idea that women prefer more masculine males in order to confer heritable immunity on their offspring. According to this view, women in reproductive age prefer average levels of masculinity (Scott, Pound, Stephen, Clark, & Penton-Voak, 2010; Stephen et al., 2012) which provides higher genetic benefits to their offspring (Foo, Simmons, & Rhodes, 2017; Lie, Rhodes, & Simmons, 2008).

### Fertility Influence on Preferences

Women’s fertility influences their preferences toward men’s faces of varying masculinity; however, these preferences differ vastly between participants, and results differ between studies (DeBruine, Jones, Smith, & Little, 2010; Feinberg, DeBruine, Jones, & Little, 2008), although some researchers did not find any robust shift in women’s mate preferences (Wood, Kressel, Joshi, & Louie, 2014). Several studies found supporting evidence that mating preferences vary depending on hormonal fluctuations in women’s menstrual cycle (Gangestad & Thornhill, 2008; Gangestad, Thornhill, & Garver, 2002; Haselton & Gangestad, 2006; Johnston, Hagel, Franklin, Fink, & Grammer, 2001; Jones et al., 2005; Lukaszewski & Roney, 2009, for a review, see Jones et al., 2008). Also, post-pubescent girls show stronger preference for men’s facial masculinity than pre-pubescent and post-menopausal ones, which further suggests that reproductive hormones are involved in facial preferences toward masculinity (Little et al., 2010; Provost, Troje, & Quinsey, 2008; Sacco, Jones, DeBruine, & Hugenberg, 2012).

It is possible that women who are more oriented toward short-term mating contexts pay more attention to masculinity as it might be correlated with men’s health (Rantala et al., 2012; Thornhill & Gangestad, 2006). The results of recent studies are mixed, however. Some researchers have shown that women with higher sociosexuality, defined as willingness to engage in uncommitted sexual relations (Simpson & Gangestad, 1991), strongly prefer masculine men’s faces (Boothroyd & Brewer, 2014; Burt et al., 2007; Sacco et al., 2012; Smith et al., 2009; Waynforth, Delwadia, & Camm, 2005). However, other studies did not show any relationship between facial masculinity preferences and sociosexuality (Glassenberg, Feinberg, Jones, Little, & DeBruine, 2010; Provost, Kormos, Kosakoski, & Quinsey, 2006). Among possible factors that might confound the relationships between women’s sociosexuality and men’s facial masculinity preference are differences in participant recruitment (Boothroyd & Brewer, 2014). In agreement with Boothroyd et al. (2008), we suggest that large samples coming from various environments are more representative of the general population than samples of university students. We focused our research on large, multicultural sample to contribute to the recent discussion about possible relationships between women’s sociosexuality and preferences of facial masculinity.

### Aims

In our study, we aimed to replicate findings on variation in masculinity preference among women of various age groups based on a new sample of women, enhance the existing pool of evidence on menstrual cyclical preference shifts on a new, large, and diverse sample, and, most importantly, compare preferences between groups of women of varying fertility (cycling, lactating, pregnant, and menopausal). A significant addition that we made in comparison with previous studies was controlling for participant’s age and sociosexuality.

## Method

### Participants

Women were recruited via online forums, mailing lists, and via personal communication. Responses were collected through a web-based survey, as it has been shown that online and laboratory studies of variation in preference for sexual dimorphism produce comparable patterns of results (Welling et al., 2008). Entering the study was conditioned by participant’s age (minimum age = 18 years old) and not using hormonal contraceptives, as hormonal contraception can influence women’s preference (Roberts et al., 2014; Welling, Puts, Roberts, Little, & Burriss, 2012). A total of 3720 heterosexual women completed the survey. Sexual orientation was based on the Kinsey scale (Kinsey, Pomeroy, & Martin, 1948). Only participants scoring 2 or lower were included in the study (exclusively heterosexual, predominantly heterosexual only incidentally homosexual or predominantly heterosexual, but more than incidentally homosexual). Participants reported age (in years), their current hormonal status (regularly menstruating, pregnant, lactating, post-menopausal), and their average length of the menstrual cycle and days since the beginning of the last menstrual bleeding.

### Procedure

Participants were presented with 20 slides (shown in a random order), and they selected via forced choice the more attractive of two stimuli pictures by answering the question “Which of the following faces is more sexually attractive?” The forced choice method is more appropriate for this kind of research compared with ratings of single pictures (Leivers, Simmons, & Rhodes, 2015). Each slide depicted two versions of the same facial picture modified to be more or less masculine. Individual preference for masculinity was calculated as the proportion of masculinized pictures being selected among the 20 pairs of pictures. This index varied from 0 (20 feminized pictures selected) to 1 (20 masculinized pictures selected).

### Measures

In this study, a subset of base pictures from a previous study examining correlates of men’s facial masculinity was used (Rantala et al., 2012). All pictures were taken using standard background and light conditions. Facial expression of the photographed person was neutral. All photographed men were Caucasian. Base pictures were transformed on a femininity/masculinity scale by using the linear difference between a composite (average) of 40 adult males and a composite of 40 adult females following established methods (Perrett et al., 1998). From each base picture, we created two stimuli pictures by adding or subtracting 50 percent of the difference between male and female composites to the base picture. What is crucial, these stimuli pictures within a pair differed only in the shape of the face and not in any other aspects (such as color, texture, symmetry), which can influence the choice (DeBruine et al., 2010). All manipulations were made with PsychoMorph program (Tiddeman, Burt, & Perrett, 2001) in a way consistent with earlier studies (Marcinkowska et al., 2014).

#### Sociosexuality

To assess attitudes toward sexual behavior, the Revised Sociosexual Orientation Inventory (SOI-R; (Penke & Asendorpf, 2008); Cronbach’s *α* = 0.73) was used. This is a nine-item scale which provides an overall measure of sociosexual orientation (e.g., “How many different partners have you had sexual intercourse with on one and only one occasion?” 1 = 0 partners, 9 = 20 partners and more) as well as three subdivisions: the Behavior subscale that measures the number of casual sex partners and the frequency of change in partners; the Attitude subscale that measures the participant’s disposition toward short-term sexual encounters; and the Desire subscale that measures the frequency of sexual fantasies or arousal in relation to potential mates with whom the individual is currently not in a committed relationship. A high SOI-R score indicates a propensity to engage in more short-term sexual relationships. The mean SOI score in this study was *M* = 3.21 (SD = 1.62, absolute range, 1–9).

#### Fertility Groups

Participants were divided into five fertility groups: (1) naturally menstruating women who were in the high-conception probability phase of their menstrual cycle, (2) naturally menstruating women who were in the low-conception probability phase, (3) pregnant, (4) lactating, and (5) post-menopausal women (Table 1). Within the naturally menstruating women group, based on the reverse count of days (deducting day of the cycle when completing the survey from stated average cycle length), those who were in 19–14 days prior to the next menses were defined as the high-conception probability group, and all other participants were defined as low-conception probability (Roney, Simmons, & Gray, 2011).

**Table 1:** Mean age and SOI-R scores of women in all fertility groups

Group	*M* age	SD	M SOI-R	SD	*N*
High-conception probability group	26.98	7.43	3.51	1.63	725
Low-conception probability group	25.90	7.09	3.11	1.62	2647
Pregnant	28.56	5.34	3.19	1.42	106
Lactating	28.61	6.04	3.32	1.45	85
Post-menopausal	54.13	6.06	3.47	1.54	157

### Statistical Analyses

Initially, analysis of covariance (ANCOVA) was used to test differences of masculinity preference among the five study groups. SOI and age of participants were treated as covariates. We also tested the assumption that there was no interaction between categorical and continuous predictors with homogeneity-of-slopes ANCOVA. The homogeneity-of-slopes model yielded nonsignificant results (all *p* > .14) implying that the homogeneity of regression slopes assumption was met. For the purpose of preliminary analysis, women were clustered into two groups—overall high fertility (high- and low-conception probability groups) and overall low fertility (lactating, pregnant and post-menopausal). As lactating women resume ovulating on average 32 weeks after the labor, we assumed that fertility in the lactating group was significantly lower than fertility in regularly menstruating group (Howie, McNeilly, Houston, Cook, & Boyle, 1982; Labbok, 2015). Levene’s test of homogeneity of samples showed, however, that the samples of participants involved here were unequal, *F*(4, 3715) = 4.77, *p* = .001, which prevents the use of ANCOVA (Levene, 1960). Various types of transformation of masculinity scores did not yield better results. We therefore followed recommendations of Quade (1967) and regressed the dependent variable (masculinity score) against covariates (SOI and age). Residuals from regression (dependent variable) were finally analyzed with ANOVA where five fertility groups were treated as categorical predictor. Fisher post hoc test was used for pair-wise comparison between means following Quade (1967). Effect sizes (partial *η*^2^) were calculated according to Huberty (2002), where values around 0.01 are considered small, 0.04 moderate, and 0.10 a large effect.

## Results

Mean preference for each fertility group was computed (Table 2). An ANOVA comparing mean preference among the five groups showed statistically significant differences, *F*(4, 3715) = 5.69, *p* < .001, albeit the effect size was low (partial *η*^2^ = 0.006). Planned comparisons showed that the overall high-fertility group (i.e., women in high- and low-conception probability phases) had significantly stronger preferences for masculinity compared with the overall low-fertility group (pregnant, lactating, and menopausal women), *F*(1, 3715) = 4.26, *p* < .05.

**Table 2:** Least square means in masculinity preference of all fertility groups after controlling for the influence of the covariates (SOI and age)

Fertility group	Mean masculinity preference	SE	− 95% CI	+ 95% CI	*N*
High-conception probability phase	0.53	0.009	0.51	0.55	725
Low-conception probability phase	0.53	0.005	0.51	0.54	2647
Pregnant	0.56	0.023	0.51	0.59	106
Lactating	0.50	0.025	0.45	0.55	85
Post-menopause	0.39	0.024	0.34	0.44	157

**Fig. 1:**
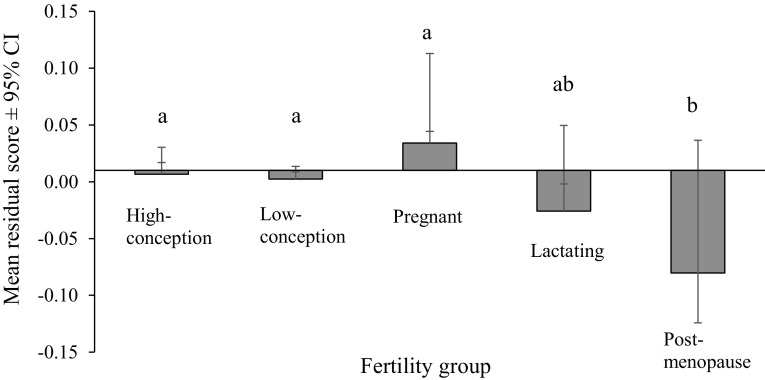
Differences in masculinity preferences (residual score controlled for age and SOI) among five fertility groups of women. Letters above bars denote differences between means based on Fisher post hoc test (*a* vs. *ab*, ns; *ab* vs. *b*, ns; *a* vs. *b*, *p* < .0001)

Masculinity preferences differed significantly among the five fertility groups (Fig. 1). Fisher post hoc test showed that women who were in both the fertile and non-fertile phases of the menstrual cycle and pregnant women had a significantly higher masculinity preference score than the post-menopausal women (all *p* < .0001). Lactating women showed no significantly different preferences than all other fertility groups of women. Other differences were not statistically significant (all *p* > .07). Both SOI score and age positively correlated with masculinity preference (Spearman *r* = .17 and .23, both *p* < .0001, respectively). When both SOI and masculinity preferences were controlled for age, correlation between these variables was low, albeit statistically significant (Spearman *r* = .14, *p* < .0001).

## Discussion

Consistent with previous studies, our results showed that current fertility status of women influenced their preference for sexual dimorphism in men’s faces, although the effect sizes were low. We found that masculinity preference of women who were naturally cycling at the time of completing the survey was stronger than that of women whose actual fertility status would prevent conceiving (post-menopausal). This finding follows a general assumption that higher probability of conceiving is related to higher preference for masculinity, because this allows women to obtain good genes for their offspring (Feinberg et al., 2006; Gangestad & Thornhill, 2008; Penton-Voak & Perrett, 2000; Penton-Voak et al., 1999; however, see Havlíček, Cobey, Barrett, Klapilova, & Roberts, 2015).

On the other hand, preferences for masculinity among fertile women were low (values about 0.5) which provides support for preferences of average levels of masculinity (Boothroyd et al., 2017; Scott et al., 2010; Stephen et al., 2012). There are at least three explanations for this finding. First, average, but not masculine male faces are cues of major histocompatibility complex (MHC) heterozygosity that is linked to immunocompetence (Lie & Simmons, 2008) and better perceived health (Foo et al., 2017). Second, it may be that our measure of masculinity preference was too narrow, because men’s masculinity is expressed not only in faces, but in a number of additional physical features like voice (Cartei, Bond, & Reby, 2014; Feinberg et al., 2008), putative male pheromones (Saxton, Lyndon, Little, & Roberts, 2008), and bodies (Little, Jones, & Burriss, 2007). Little et al. (2011) showed, however, significant consistency in women’s preferences for masculinity across all mentioned stimulus types, which suggests that this explanation is less likely. Third, men’s masculinity is associated with sexual aggression toward women (Lackie & de Man, 1997), lower trustworthiness (Smith et al., 2009), unrestricted sociosexuality (Boothroyd et al., 2008), and reduced paternal investment (Boothroyd et al., 2007). Thus, possible benefits from mating with masculine men are traded against costs associated with men’s masculinity which may result in lower preference of masculine men’s faces by women.

In our study, we did not find a difference in preferences between women in high-conception and low-conception probability phases, as found in a few previous studies (Harris, 2011, 2013; Peters, Simmons, & Rhodes, 2009). It has been proposed that there are pronounced differences in women’s preferences depending on conception probability, based on their hormonal state (for a meta-analysis, see Gildersleeve, Haselton, & Fales, 2014). Preference for masculinity and good genes was proposed to be highest around ovulation when the conception is most likely (Gangestad & Thornhill, 2008). In contrast, a preference for increased paternal investment would increase during the low-conception period, and especially during the luteal phase, when the hormonal profile somewhat resembles a beginning of pregnancy (Jones et al., 2005). We did not find a support for the lowered masculinity preference among women in their low-conception probability phase. We ascertain that the backwards cycle day counting method used in this sample was not precise enough to actually allow us to classify correctly women into low- and high-conception phase and more objective indicators of cycle status are required (Gangestad et al., 2016). It has also been suggested that participants can recall the dates of the menses onset in a faulty manner (Lukaszewski & Roney, 2009) and, most importantly, hormonal levels in menstrual cycles vary among women and among cycles of a single woman (Jasienska & Jasienski, 2008). This means that when the cycle day counting method is used, some women classified as being in “non-fertile” cycle phase may have higher levels of ovarian steroid hormones than women classified as being in “fertile” cycle phase.

In addition, regularly cycling women often have cycles that are unovulatory or cycles with low progesterone levels (Ellison, 2003; Jasienska, 2013); thus, these women, in fact, should be classified as “non-fertile” regardless of cycle phase. Due to the large number of participants and being an Internet-based study, we were unable to use methods for detecting ovulation or to measure levels of hormones. Differences between high- and low-fertility phases can be very subtle and could be better tracked by a within-participant design, rather than a between-participant one. It is close to unachievable, however, due to methodological obstacles to facilitate a within-subject design in such large data samples. We believe that between-subject, grand scale studies complement within-subject smaller sample studies. Hence, the lack of cyclical shift in masculinity preference in our data does not exclude theory that there is a difference between women of varying fertility status.

As pregnancy and menopause signal a long-term state of non-fertility, we could expect that from an evolutionary point of view women’s preference should be directed to resources and parenting skills, rather than good genes (Cobey, Little, & Roberts, 2015; Little et al., 2010). Preferences of post-menopausal women for more feminine men’s faces could be caused by a shift from mating-oriented behavior to family-oriented behavior (Hawkes, O’Connell, Jones, Alvarez, & Charnov, 1998). More feminine men have apparently lower testosterone levels (Schaefer, Fink, Mitteroecker, Neave, & Bookstein, 2005) that can be associated with higher involvement in paternal care (Muller et al., 2009). It may be that after menopause, a woman’s preference may change toward better parental and/or grandparental care (Rantala, Polkki, & Rantala, 2010).

Cobey et al. (2015) found that postpartum women (up to 12 weeks after birth) showed lower masculinity preference than pregnant women. Similarly, we found that pregnant women showed stronger preference for masculinity compared with lactating women albeit the difference was short of statistical significance (Fisher post hoc test, *p* = .077), perhaps because our sample consisted of exclusively breastfeeding women. This difference, albeit not statistically significant, could be explained by hormonal changes associated with transition to parenthood, during which baseline testosterone level is decreasing (Kuzawa, Gettler, Huang, & McDade, 2010). Indeed, Alder, Cook, Davidson, West, and Bancroft (1986) found that testosterone and androstenedione levels were significantly lower in lactating women who reported severe reduction in sexual interest. Such physiological change would be adaptive, because lowered attraction to men’s facial cues associated with sexual attractiveness may enhance maternal behavior (Cobey et al., 2015).

Notably, our results could stem from the reproductive ambition of participants (i.e., desire to become pregnant), which is positively correlated with preference for masculinity in men’s faces (Watkins, 2012). It is possible that reproductive ambition would not change over the cycle but rather result from the reproductive history of a woman—hence, significant difference between cycling and not cycling women, and a lack of difference between high- and low-conception probability phases.

One possible confounding factor in our research could be men’s age on facial stimuli, because the age itself changes and is related to preferences for partners as well. Buss and Schmitt (1993), in their classic paper on mating preferences, showed that women in 37 cultures preferred older men. The age of a preferred man was on average 3.5 years older than the age of a woman. We cannot exclude the possibility that preferences for masculinity were confounded by higher age differences between older, post-menopausal women and male facial stimuli. Older women, however, showed similar masculinity preferences as lactating women and both groups show similar androgen decline (Alder et al., 1986; Davison, Bell, Donath, Montalto, & Davis, 2005) supporting the idea that women’s long-term hormonal changes influences mating preferences (Havlíček et al., 2015; Little et al., 2010). Future research can examine whether age differences between raters and facial stimuli influence mating preferences.

Several studies showed that women’s sociosexuality was positively associated with preferences for masculine men’s faces (e.g., Burt et al., 2007; Waynforth et al., 2005). The present study confirmed this relationship, but the correlation was very weak. Most possibly, these associations are influenced by several other variables that were not controlled in this study. More attractive women show, for example, higher sociosexuality (Clark, 2004) and stronger preferences for masculine male faces (Little, Burt, Penton-Voak, & Perrett, 2001). Unpartnered women showed higher sociosexuality scores that significantly correlated with preference for men’s facial masculinity compared with partnered women (Sacco et al., 2012). Some personality traits, such as extraversion, correlate with women’s sociosexuality (Wright & Reise, 1997) and, in turn, extraversion was found to correlate with women’s preferences for masculinity in men’s faces (Welling, DeBruine, Little, & Jones, 2009). Future research on sociosexuality and masculinity preferences should take more factors influencing masculinity preference into account before firm conclusions can be made.

### Conclusions

To conclude, we found an effect of overall fertility status on facial sexual dimorphism preference in women. It appears that the overall lowered fertility state caused by menopause affects the masculinity preference. Preferences for masculinity in naturally cycling women were, however, low, which can be explained by preferences for average, rather than masculine faces that provide health benefits to children. We did not find differences in masculinity preference depending on varying conception probability throughout the menstrual cycle though (based on the backward counting days method). Women’s sociosexuality showed positive, but very weak influence on preferences for masculine men’s faces. Based on our results, we suggest that women’s long-term hormonal state is a stronger predictor of preference for sexual dimorphism than changes in hormonal levels throughout the cycle.
